# Antenna Modification Leads to Enhanced Nitrogenase Activity in a High Light-Tolerant Cyanobacterium

**DOI:** 10.1128/mbio.03408-21

**Published:** 2021-12-21

**Authors:** Anindita Bandyopadhyay, Zi Ye, Zuzana Benedikty, Martin Trtilek, Himadri B. Pakrasi

**Affiliations:** a Department of Biology, Washington University, St. Louis, Missouri, USA; b Photon Systems Instruments, Drasov, Czech Republic; c State Key Laboratory of Freshwater Ecology and Biotechnology, Institute of Hydrobiology, Chinese Academy of Sciences, Wuhan, China; University of Washington

**Keywords:** Cyanobacterium, cyclic electron flow, heterocyst, nitrogenase, photosystem I, phycobilisome

## Abstract

Biological nitrogen fixation is an energy-intensive process that contributes significantly toward supporting life on this planet. Among nitrogen-fixing organisms, cyanobacteria remain unrivaled in their ability to fuel the energetically expensive nitrogenase reaction with photosynthetically harnessed solar energy. In heterocystous cyanobacteria, light-driven, photosystem I (PSI)-mediated ATP synthesis plays a key role in propelling the nitrogenase reaction. Efficient light transfer to the photosystems relies on phycobilisomes (PBS), the major antenna protein complexes. PBS undergo degradation as a natural response to nitrogen starvation. Upon nitrogen availability, these proteins are resynthesized back to normal levels in vegetative cells, but their occurrence and function in heterocysts remain inconclusive. *Anabaena* 33047 is a heterocystous cyanobacterium that thrives under high light, harbors larger amounts of PBS in its heterocysts, and fixes nitrogen at higher rates compared to other heterocystous cyanobacteria. To assess the relationship between PBS in heterocysts and nitrogenase function, we engineered a strain that retains large amounts of the antenna proteins in its heterocysts. Intriguingly, under high light intensities, the engineered strain exhibited unusually high rates of nitrogenase activity compared to the wild type. Spectroscopic analysis revealed altered PSI kinetics in the mutant with increased cyclic electron flow around PSI, a route that contributes to ATP generation and nitrogenase activity in heterocysts. Retaining higher levels of PBS in heterocysts appears to be an effective strategy to enhance nitrogenase function in cyanobacteria that are equipped with the machinery to operate under high light intensities.

## INTRODUCTION

Nitrogen fixation is a crucial process by which molecular nitrogen is rendered accessible to all life forms. The process is energy-intensive, making fixed nitrogen a sparse commodity in many natural habitats and cultivated lands. Of the nitrogen-fixing organisms, photosynthetic prokaryotes with the inherent ability to couple the energy-demanding nitrogenase reaction with energy-generating photosynthesis have a distinct advantage. Cyanobacteria constitute a group of oxygenic photosynthetic prokaryotes, some members of which have the dual ability to fix carbon and nitrogen. Nitrogenase, the enzyme catalyzing microbial nitrogen fixation is prone to destruction by oxygen, and, as such, diazotrophic cyanobacterial species have evolved to separate the incompatible processes of photosynthesis and nitrogen fixation temporally or spatially.

Some filamentous cyanobacteria segregate nitrogen fixation in specialized cells called heterocysts. Heterocysts lack functional photosystem II (PSII) and oxygenic photosynthesis but have a fully functional photosystem I (PSI) that is involved in generating ATP by cyclic photophosphorylation (1). Studies have indicated that the main source of ATP for nitrogen fixation in heterocysts is the light reactions in the thylakoids and that the required ATP can be provided entirely by light-driven cyclic photophosphorylation (2–4). Compared to adjoining vegetative cells, heterocysts have a significantly higher abundance of PSI protein subunits (5, 6) as well as subunits of the Cyt-b_6_f complex involved in cyclic electron transport (3, 7). In addition, the number of photosynthetic units (ratio of chlorophyll a/P700) per heterocyst is 50% higher than vegetative cells (2, 7–8). All these pieces of evidence point to a distinct role for light and PSI in heterocyst function.

Light harvest in cyanobacteria is achieved by large protein complexes called phycobilisomes (PBS) that transfer energy to PSI and PSII reaction centers (9). PBS undergo degradation as an adaptive strategy to various environmental stress conditions like high light and nitrogen deficiency (10, 11). Although the mechanism of PBS degradation is yet to be fully elucidated, it has been established that NblA, a small protein consisting of 60 amino acids is a key player in the process (12). The effect of *nblA* deletion has been extensively investigated in nondiazotrophic model strains of cyanobacteria in which PBS degradation is an essential adaptive mechanism to survive high light and nitrogen stress (13–15). In contrast, PBS degradation is less likely to have a role in acclimation to these stress factors in high light-tolerant, diazotrophic cyanobacteria and the effect of *nblA* deletion in these strains has yet to be investigated.

*Anabaena* sp. ATCC 33047 is a fast-growing filamentous cyanobacterium that has garnered interest for its ability to thrive under very high light intensities and fix carbon and nitrogen at high rates (16). This strain was shown to harbor significant amounts of PBS in its heterocysts, implying expensive resynthesis of these antenna protein complexes after the initial nitrogen acclimation phase. However, the function of these proteins in mature heterocysts and their relation to nitrogen fixation was not obvious (17). Earlier studies implicating a role for PBS in nitrogen fixation, specifically in energy transfer to PSI (7, 18, 19), led us to develop a genome engineering strategy for this previously recalcitrant strain and generate a Δ*nblA* mutant that would enable us to investigate the effect of heterocyst antenna modification on nitrogenase function. The Δ*nblA* mutant retained high amounts of PBS in its heterocysts and exhibited 2 to 3-fold higher nitrogenase activity compared to the wild-type (WT). Spectroscopic analysis of the mutant indicated higher cyclic electron flow, possibly resulting from higher energy transfer to PSI, facilitated by excess PBS in heterocysts. This in turn leads to higher ATP generation and enhanced nitrogenase activity. Our study suggests that augmenting light capture by heterocysts of high light-tolerant cyanobacteria can be an effective strategy to enhance nitrogen fixation.

## RESULTS

### *Anabaena* 33047 thrives under very high light intensities.

**FIG 1:**
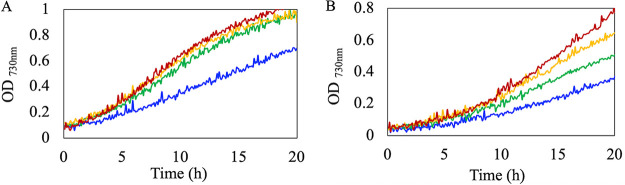
*Anabaena* 33047 thrives under high light. Representative growth curves of WT cells under different light intensities (blue, 500; green, 1000; yellow, 1500; red, 2000 μmol m^−2^ s^−1^). Cells were grown at 42°C in a medium with (A) or without (B) added nitrogen sources. Cultures were supplemented with 1% CO_2_.

*Anabaena* 33047 exhibited rapid growth both in the presence and absence of added nitrogen sources and an increase in growth rate was observed with increasing light intensity (Fig. 1A and B). When grown at 42°C in media supplemented with nitrogen sources and 1% CO_2_, the highest growth rate (doubling time of 3.18±0.16 h) was observed under 1500 to 2000 μmol photons m^−2^s^−1^ of light (Fig. 1A). At higher light intensities (up to 3000 μmol photons m^−2^s^−1^), although no increase in growth rate was observed, higher biomass accumulation could be achieved. When grown under light intensities of 3000 μmol photons m^−2^s^−1^, high amounts of exopolysaccharide (EPS) were secreted into the medium, which led to excessive clumping of filaments (data not shown). When grown under light intensities of 2000 μmol photons m^−2^s^−1^ in nitrogen-deplete medium, a doubling time of 3.8±0.43 h was observed (Fig. 1B). These growth rates are considerably higher than the commonly studied model filamentous strain *Anabaena* 7120, which, under 400 μmol photons m^−2^s^−1^ at 28°C, exhibited a doubling time of 8.5±0.32 h under N_2_-sufficient and 12.2±0.86 h under N_2_-deficient conditions (Fig. S2). Under light intensities >1500 μmol photons m^−2^s^−1^, this strain exhibited growth inhibition (data not shown). In contrast, the growth of *Anabaena* 33047 was greatly inhibited under light intensities <500 μmol photons m^−2^s^−1^.

10.1128/mbio.03408-21.2 FIG S2 Representative growth curves showing a comparison of WT *Anabaena* 7120 grown under 400 μmol m^−2^s^−1^ light intensities at 28°C in medium with (blue) and without (green) sources of fixed nitrogen and supplemented with 1% CO_2_. Download FIG S2, PDF file, 0.10 MB. Copyright © 2021 Bandyopadhyay et al. 2021 Bandyopadhyay et al. https://creativecommons.org/licenses/by/4.0/ This content is distributed under the terms of the Creative Commons Attribution 4.0 International license.

Light microscopic analysis of filaments grown under nitrogen-fixing conditions revealed higher frequency (14%) of heterocysts in *Anabaena* 33047 compared to *Anabaena* 7120 (10%) (Fig. S1A) and about 50% higher specific activity of the nitrogenase enzyme when the cells were grown under conditions optimal (see Methods and Materials) for *Anabaena* 7120 (Fig. S1B).

10.1128/mbio.03408-21.1 FIG S1 Characterization of *Anabaena* 33047. (A) Comparison of bright field and fluorescence images of filaments of *Anabaena* 33047 and *Anabaena* 7120 grown under nitrogen-fixing conditions. A higher frequency of heterocysts is observed in *Anabaena* 33047 (arrows). (B) Comparison of nitrogenase activity in *Anabaena* 33047 and *Anabaena* 7120. Representative data are shown as the averages of three biological replicates, and error bars show the standard deviations (SDs) from the averages. Download FIG S1, PDF file, 0.5 MB. Copyright © 2021 Bandyopadhyay et al. 2021 Bandyopadhyay et al. https://creativecommons.org/licenses/by/4.0/ This content is distributed under the terms of the Creative Commons Attribution 4.0 International license.

### Engineering *Anabaena* 33047 for genetic amenability.

As anticipated, *the Anabaena* 33047 strain procured from the University of Texas at Austin (UTEX) culture collection proved to be intractable to all targeted genetic modification attempts. Studies have documented the successful transformation of filamentous cyanobacteria by modification of the host restriction-modification (RM) system (20). None of the existing conjugal, helper, or suicide cargo plasmids (21) yielded success with the conjugation of *Anabaena* 33047. We mined the genome sequence of this strain to ascertain its RM system and subsequently modify it to our advantage. We identified several methylase or methyltransferase genes that appeared to be associated with a type II restriction system (adjacent genes encode restriction enzymes). These genes, together with their upstream regions, were cloned into a helper plasmid (based on helper plasmid pRL623) in various combinations and tested for conjugation efficiency. Various conjugation experiments revealed that a helper plasmid with five genes (see Methods and Materials) in tandem, driven by a lac promoter, was the most effective at conjugating *Anabaena* 33047 (Fig. S3A). Using the newly synthesized plasmid, we performed targeted gene deletions and successfully generated a Δ*nblA* mutant of this strain. Surprisingly, unlike other filamentous cyanobacterial strains where single homologous recombination is known to be prevalent in the first few generations, almost all colonies tested for the gene deletions in *Anabaena* 33047 were double recombinants in their first generation (Fig. S3B). The complete segregation of the mutant was verified by PCR analysis (Fig. S3C).

10.1128/mbio.03408-21.3 FIG S3 Engineering genetic amenability in *Anabaena* 33047. (A) Schematics of the strategy used for designing the helper plasmid that was used in tri-parental conjugation of *Anabaena* 33047. (B) Segregation analysis of the Δ*nblA* mutant. (C) PCR analysis verifying segregation of the Δ*nblA* mutant. Download FIG S3, PDF file, 0.2 MB. Copyright © 2021 Bandyopadhyay et al. 2021 Bandyopadhyay et al. https://creativecommons.org/licenses/by/4.0/ This content is distributed under the terms of the Creative Commons Attribution 4.0 International license.

### The *ΔnblA* mutant retained large amounts of PBSs in heterocysts.

**FIG 2:**
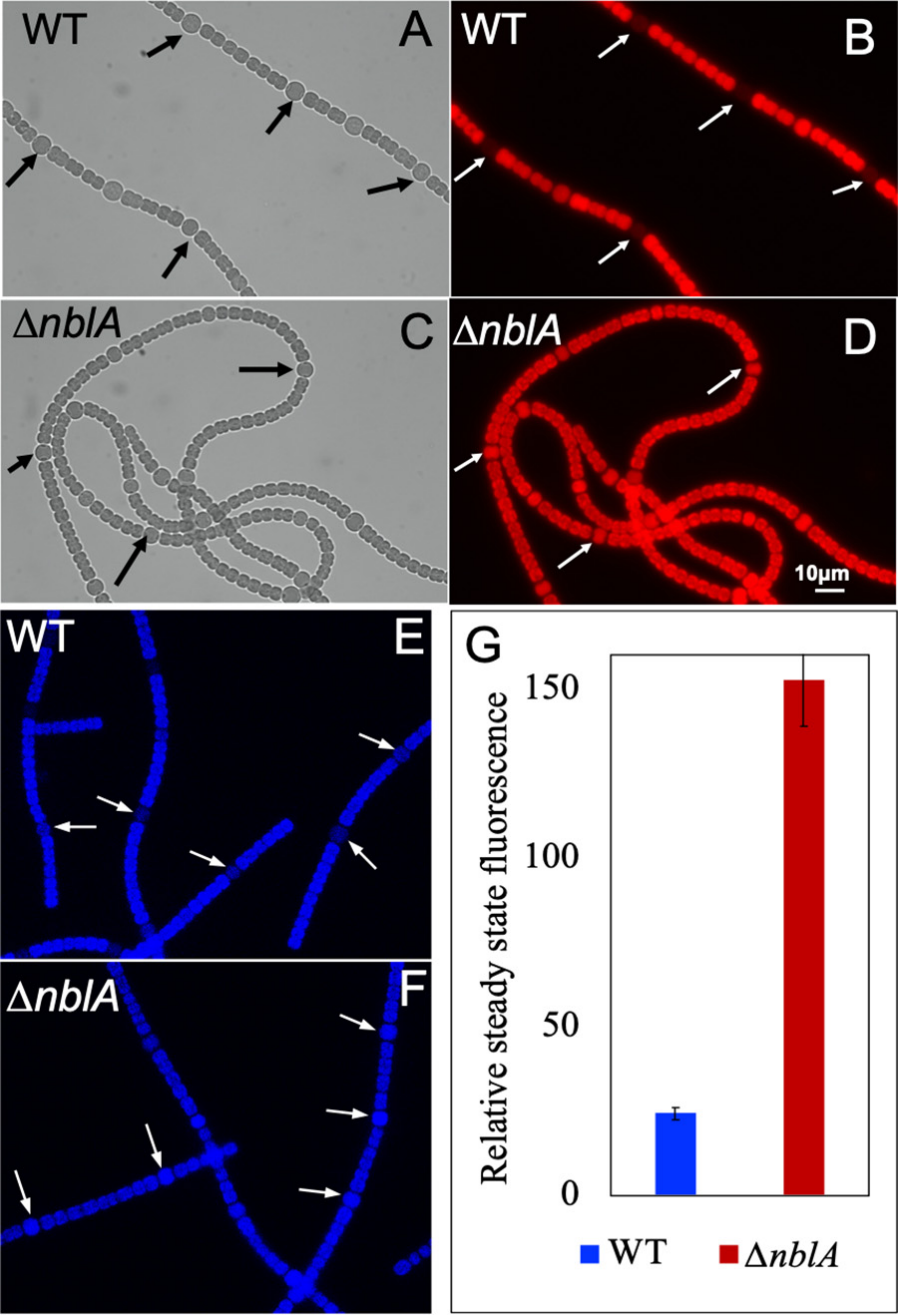
Microscopic analysis showing large amounts of PBS in heterocysts of the Δ*nblA* mutant of *Anabaena* 33047. (A to D) Brightfield and fluorescence microscopic images of WT (A and B) and Δ*nblA* (C and D) mutant of *Anabaena* 33047 grown in media lacking fixed nitrogen sources. A large amount of PBS was retained in the Δ*nblA* heterocysts compared to the WT (arrows). (E to G) FKM analysis of WT and Δ*nblA* heterocysts. (E and F) The bright signal was observed in heterocysts of the Δ*nblA* mutant compared to the WT (arrows). (G) Average steady-state fluorescence obtained from WT and Δ*nblA* mutant after excitation of phycobilisomes in heterocysts.

Fluorescence microscopic analysis revealed that, under nitrogen-deficient growth conditions, PBSs returned to their normal levels in the vegetative cells of the WT within 24h of onset of nitrogen starvation. However, the PBS content of the heterocysts remained significantly lower. In contrast, the Δ*nblA* strain exhibited high amounts of PBS both in the vegetative cells, and the heterocysts as the filaments acclimatized to nitrogen deficiency and both cell types appeared brightly fluorescent (Fig. 2A to D). We quantitated the PBS content in the mutant and WT heterocysts with the help of a fluorescence kinetic microscope (FKM) (Fig. 2E and F). Steady-state fluorescence depicting the number of phycobiliproteins in the heterocysts was about 8-fold higher in the Δ*nblA* mutant compared to that in the WT (Fig. 2G). In contrast, the PBS levels in the vegetative cells of a nitrogen-fixing filament were not significantly different between the WT and the mutant strains (data not shown).

To evaluate the effect of averting PBS degradation on the physiology of the fast-growing, high light-tolerant cyanobacterium, we compared the growth rate of the WT and the Δ*nblA* mutant under different light intensities. Differences in growth rate were observed between the mutant and the WT under low light intensities. When grown under 250 μmol photons m^−2^s^−1^, the mutant exhibited ∼50% higher growth rate compared to the WT (Fig. S4). The difference in growth rate narrowed with increasing light intensities and, at 500 μmol photons m^−2^s^−1^, the mutant grew ∼12% faster than the WT (Fig. S4). The mutant and the WT exhibited comparable growth rates up to 1500 μmol photons m^−2^s^−1^. Under higher light intensities (≥2000 μmol photons m^−2^s^−1^), the mutant initially grew at a slightly reduced rate compared to the WT, although the final biomass accumulation was similar in both the strains (Fig. S4). The higher growth rate under low light correlated with increased quantum yield and enhanced oxygen evolution rates in the mutant (data not shown), which indicated higher photosynthetic activity. In contrast, photosynthetic activity under high light was slightly reduced.

10.1128/mbio.03408-21.4 FIG S4 Representative curves showing growth comparison of WT (solid lines) and Δ*nblA* (dotted lines) strains of *Anabaena* 33047 under low and high light intensities at 42°C in medium without sources of fixed nitrogen and supplemented with 1% CO_2_. Red, 2000 μmol m^−2^ s^−1^; green, 500 μmol m^−2^ s^−1^; gray, 250 μmol m^−2^ s^−1^. Download FIG S4, PDF file, 0.05 MB. Copyright © 2021 Bandyopadhyay et al. 2021 Bandyopadhyay et al. https://creativecommons.org/licenses/by/4.0/ This content is distributed under the terms of the Creative Commons Attribution 4.0 International license.

### The Δ*nblA* mutant exhibits unusually high rates of nitrogenase activity.

**FIG 3:**
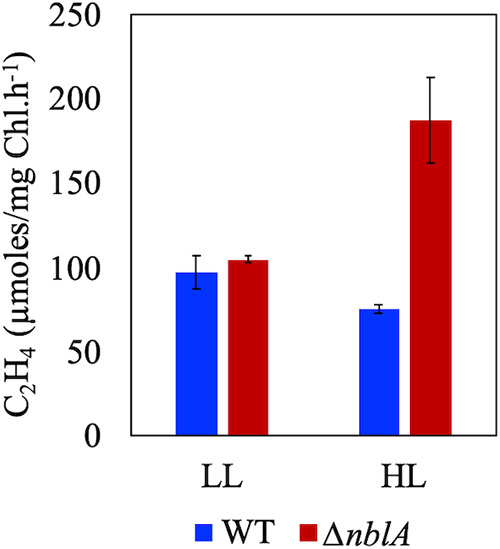
Nitrogenase activity in the WT and Δ*nblA* strains of *Anabaena* 33047 grown in nitrogen deplete medium under low (250 μmol photons m^−2^s^−1^) or high (2000 μmol photons m^−2^s^−1^) light intensities. Representative data are shown as the average of three biological replicates, and error bars show the standard deviation from the average.

To assess the effect of PBS retention on heterocyst function in the high light-tolerant *Anabaena* 33047, we compared nitrogenase activity in the WT and Δ*nblA* strains. When grown under 250 μmol photons m^−2^s^−1^ light, no significant difference in nitrogenase activity was observed between the mutant and the WT (Fig. 3). However, when grown under 2000 μmol photons m^−2^s^−1^, the mutant exhibited 2 to 3-fold higher specific rates of nitrogenase activity compared to the WT (Fig. 3). If grown for more than 36 h under high light in nitrogen deplete medium, both the mutant and the WT filaments clumped into a ball due to excess EPS secretion, and both strains exhibited greatly reduced rates of nitrogenase activity. When incubated in the dark, the rates of nitrogenase activity were drastically reduced for both the mutant and the WT grown under low or high light intensities (data not shown), indicating that the activity is light-dependent. When treated with DBMIB (2,5-dibromo-3-methyl-6-isopropyl benzoquinone), a quinone analog that inhibits the cytochrome b_6_f complex and, thus, disables cyclic electron flow (22), nitrogen fixation rates were drastically reduced both in the mutant and the WT. On the other hand, incubation with DCMU (3-[3,4-dichlorophenyl]-1,1-dimethylurea), which blocks PSII and linear electron transport alone, did not have an inhibitory effect on nitrogenase activity in the WT or the mutant (Fig. S5).

10.1128/mbio.03408-21.5 FIG S5 Nitrogenase activity in the WT and Δ*nblA* strains of *Anabaena* 33047 grown in nitrogen deplete medium and assayed in the presence of the inhibitors DCMU (10 μM) and DBMIB (1 μM). Representative data are shown as the average of three biological replicates, and error bars show the standard deviation from the average. Download FIG S5, PDF file, 0.05 MB. Copyright © 2021 Bandyopadhyay et al. 2021 Bandyopadhyay et al. https://creativecommons.org/licenses/by/4.0/ This content is distributed under the terms of the Creative Commons Attribution 4.0 International license.

### P700 oxidation kinetics suggest higher cyclic electron flow in the mutant.

**FIG 4:**
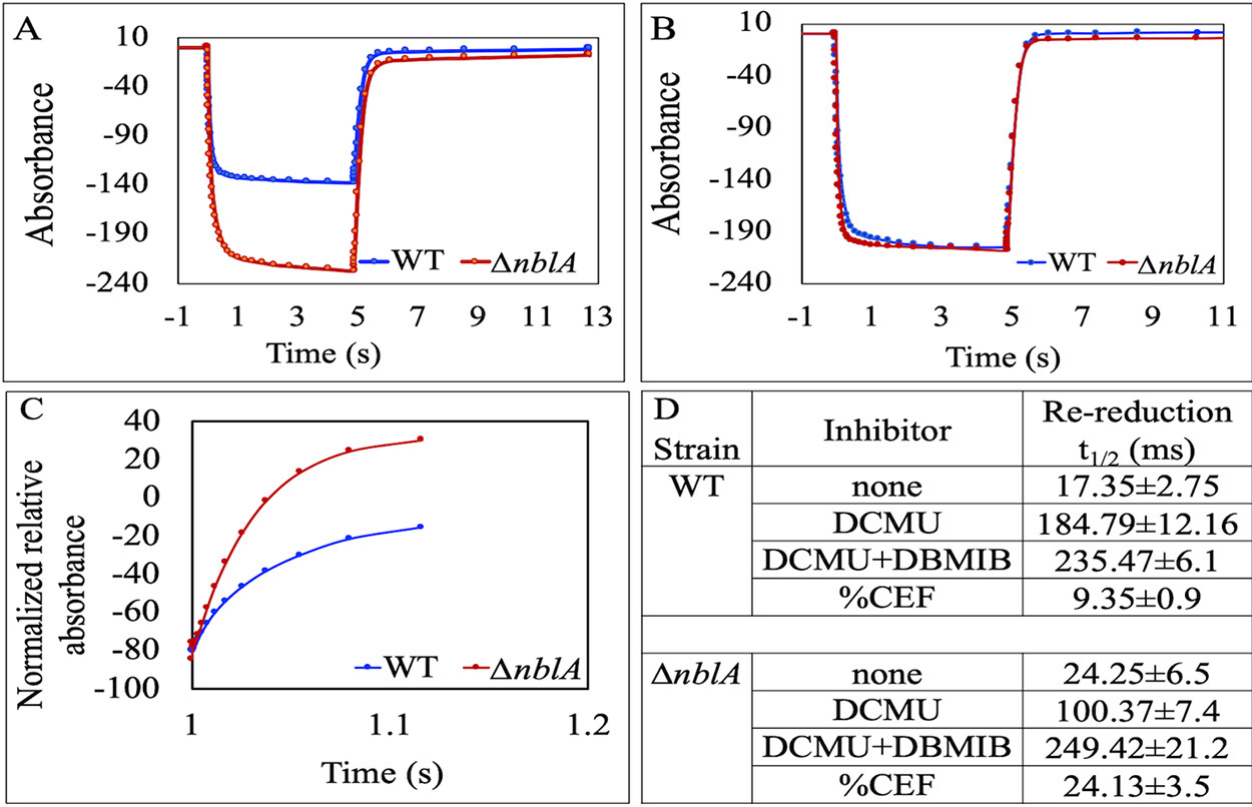
P700 redox kinetics for WT and Δ*nblA* mutant of *Anabaena* 33047 grown under high light (2000 μmol photons m^−2^s^−1^). Dark-adapted cells were exposed to a pulse of actinic light for 5 s (white bar above [A]). This was followed by dark incubation (black bar above [A]). During this time course, measuring flashes of 705 nm light probed the redox state of the P700 reaction center of PSI. (A) P700 redox kinetics in DCMU treated (10 μM) WT and Δ*nblA* cells. (B) P700 redox kinetics in WT and Δ*nblA* cells treated with DCMU (10 μM) and DBMIB (1 μM). (C) Details of the re-reduction kinetics of P700^+^ in the dark (normalized to total oxidizable P_700_) for the experiment in panel A. Each trace is an average of 3 independent experiments. (D) The halftimes (milliseconds) for re-reduction of P700^+^ in the dark.

To investigate the basis of the enhanced nitrogenase activity in the Δ*nblA* mutant grown under high light and to assess if the mutant exhibited any difference in PSI function, we probed the oxidation/reduction kinetics of PSI reaction centers in both the mutant and the WT using a Joliot type JTS-10 spectrophotometer (23, 24). We exposed dark-adapted WT and Δ*nblA* cells grown under high light to a 5 s pulse of actinic light and measured the oxidation of P700. This was followed by the measurement of the reduction kinetics in the dark. These measurements were carried out in the absence and presence of the inhibitor DCMU (Fig. S6A and Fig. 4), which blocks PSII and linear electron flow in the vegetative cells, thus allowing only cyclic electron flow (CEF) around PSI in the vegetative cells and heterocysts. We also assessed samples treated with DCMU and DBMIB, inhibiting both linear and cyclic electron flows.

10.1128/mbio.03408-21.6 FIG S6 P700 redox kinetics for WT and Δ*nblA* mutant of *Anabaena* 33047 grown under high light (2000 μmol photons m^−2^s^−1^). (A) P700 kinetics in the WT and Δ*nblA* mutant in the absence of any inhibitor. (B) The details of the re-reduction kinetics of P700^+^ in the dark (normalized to total oxidizable P_700_) for the experiments in Fig. 4B. (C) P700 kinetics in the WT and Δ*nblA* mutant grown under nitrogen sufficient conditions and assayed in the presence of DCMU (10μM). Each trace is an average of 3 independent experiments. Download FIG S6, PDF file, 0.2 MB. Copyright © 2021 Bandyopadhyay et al. 2021 Bandyopadhyay et al. https://creativecommons.org/licenses/by/4.0/ This content is distributed under the terms of the Creative Commons Attribution 4.0 International license.

When linear electron flow was blocked with DCMU, the P700 oxidation kinetics between the WT and the mutant filaments grown under nitrogen-fixing conditions were strikingly different with greater oxidation of P700 achieved in the Δ*nblA* mutant compared to the WT (Fig. 4A). In contrast, when treated with DCMU and DBMIB, the oxidation of P700 between the two strains was similar (Fig. 4B). This indicated that the difference observed with DCMU treatment could be a result of an enhanced flow of electrons to PSI in the mutant via the cyclic pathway, which is obstructed by DBMIB treatment and is not a true reflection of the amount of P700 in the cells. To enable comparison between the mutant and the WT, we normalized the kinetics of P700^+^ re-reduction in the dark (Fig. 4C) to the maximal oxidation observed (24). The Δ*nblA* mutant exhibited faster re-reduction kinetics in the presence of DCMU compared to the WT (Fig. 4C). In contrast, the re-reduction kinetics appeared to be similar in the two strains when treated with DCMU and DBMIB (Fig. S6B). Based on the assumption that CEF is the main contributor to P700^+^ re-reduction, we calculated the percentage contribution of cyclic versus linear electron transport to P700^+^ re-reduction in the WT and mutant filaments (Fig. 4D) (24, 25). When cells grown under high light were treated with DCMU, the mutant showed greater reliance on CEF for P700 oxidation compared to the WT. Under these conditions, the cyclic process accounted for ∼24% electron flow to P700^+^ in the mutant (Fig. 4D). To assess the contribution of vegetative cells to the observed difference in P700 oxidation between the mutant and the WT, we measured P700 oxidation in filaments grown under nitrogen sufficient conditions (filaments without any heterocysts). No significant difference in P700 oxidation was observed between the vegetative cells of the WT and the mutant treated with DCMU (Fig. S6C).

## DISCUSSION

*Anabaena* 33047 is unique among heterocystous cyanobacteria in its ability to thrive under high light intensities. The strain also fixed nitrogen at higher rates (Fig. S1B) and harbored a larger amount of PBS in its heterocysts compared to other heterocystous cyanobacteria (17). This study was initiated to investigate the role of and relationship between high light and PBS content in the heterocysts of *Anabaena* 33047. To this end, we successfully engineered a strategy to modify the genome of this previously recalcitrant strain and generated a *nblA* deletion mutant, which retained large amounts of PBS in its heterocysts and enabled us to assess the function of these antenna pigment proteins in nitrogen fixation.

Our current understanding of the effects of averting PBS degradation in cyanobacteria by deleting *nblA* relies largely on studies in nondiazotrophic strains (12, 13, 26–28). The only diazotrophic strain where a Δ*nblA* mutant has been characterized so far is *Anabaena* 7120 and under the conditions tested, the deletion did not have any impact on growth or nitrogen fixation (29). This indicated that degradation of PBS is not an essential adaptive strategy for heterocystous strains to transition from a nitrogen-deplete to a nitrogen-fixing condition.

Various studies revealed the functional association between PBS and PSI in heterocysts. A study in Anabaena variabilis demonstrated a role for PBS in the efficient transfer of light energy to PSI and photooxidation of P-700, the reaction center of PSI (30). A similar study in *Anabaena* 7120 reported the isolation of a PBS-PSI super complex, the spectral analysis of which revealed efficient energy transfer from PBS to PSI (19). A previously unknown linker component cpcL was shown to be involved in establishing the connection between PBS and PSI. Interestingly, this linker is found in many heterocystous cyanobacteria, including *Anabaena* 33047, but not in unicellular cyanobacteria that fix nitrogen at night. The study found that the levels of this linker were 4-fold higher in heterocysts compared to vegetative cells and proposed a role for the PBS-cpcL-PSI complex in harvesting light energy and facilitating PSI-driven nitrogen fixation. These findings were also supported by a more recent study that showed the transfer of energy from cpcL-PBS to PSI in a mutant of *Anabaena* 7120 (4). In addition, a role for these phycobiliproteins in supporting light-driven nitrogenase activity in heterocysts was also demonstrated (30).

**FIG 5:**
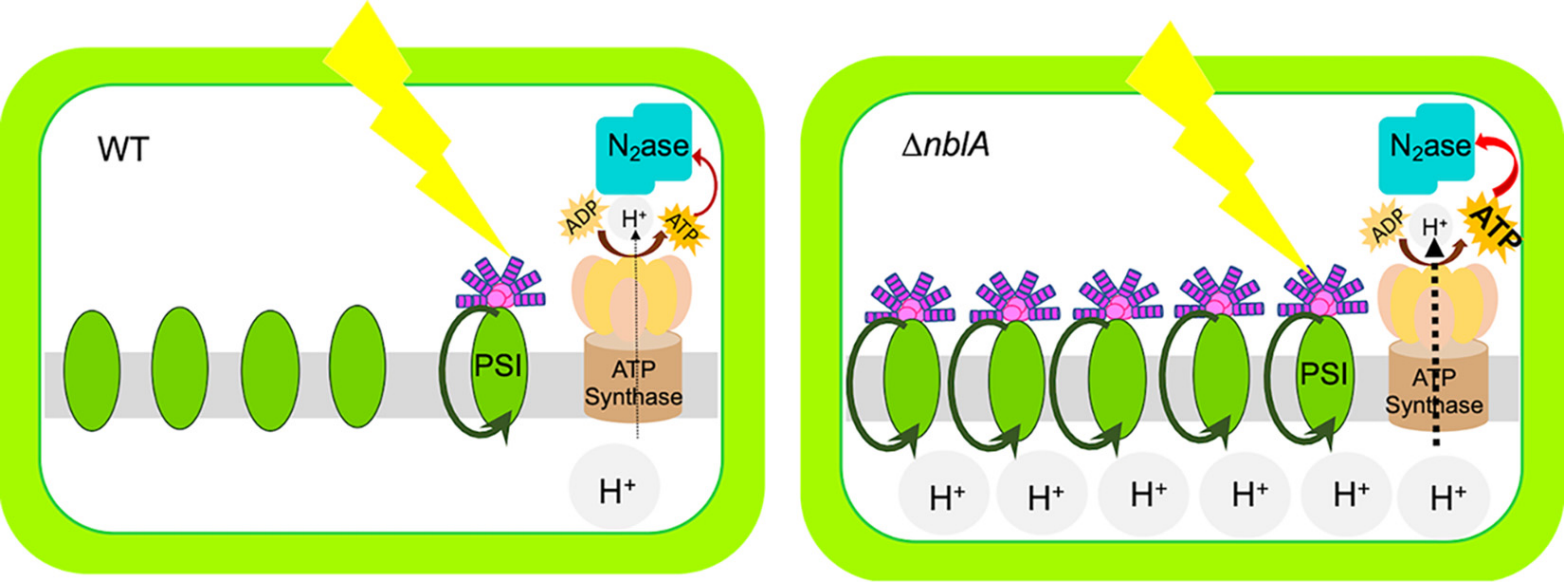
Schematics depicting the differences in the heterocysts of the WT and Δ*nblA* strains that contribute to enhanced nitrogenase activity. A higher abundance of phycobilisomes in the mutant heterocyst and their association with PSI centers leads to higher ATP generation mediated by cyclic electron flow. The ATP feeds into the nitrogenase enzyme complex leading to higher rates of nitrogen fixation in the Δ*nblA* mutant.

In heterocystous cyanobacteria, cyclic electron flow around PSI plays a crucial role in driving nitrogenase activity (31–33). Cyclic electron flow relies on the transfer of reducing equivalents from PSI to the plastoquinone pool via the NDH-1 complex. The reduced plastoquinone pool is reoxidized by the cytochrome b_6_/f (Cyt-b6/f) complex, which transfers electrons back to PSI via plastocyanin, thereby completing the cycle. This cyclic flow of electrons is coupled to the generation of a proton gradient across the thylakoid membrane, which drives ATP synthesis (Fig. 5). CEF around PSI is light-dependent and it has been demonstrated that CEF can vary with light intensity with increased irradiance leading to higher rates of CEF (34, 35).

The Δ*nblA* mutant exhibited higher PBS content in its heterocysts and higher nitrogenase activity. Spectroscopic studies demonstrated higher CEF in the mutant compared to the WT (Fig. 2 to 4). Our FKM analysis did not reveal any significant difference in pigment content between the vegetative cells of the mutant and the WT (after PBS is resynthesized in the WT) and no significant difference was observed in P700 oxidation between the vegetative cells of the mutant and the WT grown under nitrogen sufficient conditions. These observations suggest that the enhanced CEF detected in the mutant is likely a reflection of the altered photochemistry in its heterocysts brought about by higher levels of PBS, which, in turn, contributes to enhanced nitrogenase activity. In addition, averting PBS degradation can eliminate the need for the expensive resynthesis of these large antenna complexes, a phenomenon that likely takes place in the wild-type *Anabaena* 33047 heterocysts that harbor larger amounts of PBS compared to other cyanobacteria. Averting antenna degradation in the Δ*nblA* mutant possibly allows utilization of the cellular resources for the energy-intensive nitrogen fixation process instead. Retention of PBS in the vegetative cells of the mutant during acclimation to nitrogen limitation did not seem to have any significant adverse effect on growth or metabolism. A small inhibitory effect on growth was observed for a few hours after inoculation into nitrogen depleted medium, which could be a result of a slight reduction in photosynthesis observed under these conditions, but the final biomass accumulation was similar to the WT. Interestingly, the higher nitrogenase activity in the mutant did not contribute to faster growth under high light, suggesting that other cellular resources could be limiting or that the high rates of N_2_-fixation achieved by the WT are optimal to support the fast growth of this strain. The excess N_2_ in the Δ*nblA* strain is probably channelized into storage reserves. When grown for an extended period (>30h) excess EPS secretion led to clumping of the filaments into a tight ball thereby limiting light availability to the cells resulting in greatly reduced nitrogenase activity indicating that access to high light is crucial for augmenting nitrogenase function. In contrast, when grown under low light, the presence of PBS turned out to be advantageous for efficient light-harvesting in the mutant and this was reflected in faster growth compared to the WT (Fig. S4). However, the low light growth conditions could not elicit an increase in nitrogenase activity. This again suggests that the high rates of nitrogenase activity in the WT *Anabaena* 33047 are optimal to support growth under all conditions, but light can be a limiting factor. The mutant thus holds the potential to channelize the excess pool of nitrogen fixed under high light toward nitrogen-rich products of interest.

Our study demonstrates a link between PBS content and nitrogenase activity in heterocysts. Increased light absorption by PBS leads to enhanced cyclic phosphorylation, which is the driving force for nitrogen fixation. Thus, in a high light-tolerant strain like *Anabaena* 33047 modifying the heterocyst antenna by disabling degradation of PBS during nitrogen acclimation appears to be an effective strategy for enhancing nitrogen fixation rates.

## MATERIALS AND METHODS

### Cyanobacterial strains and growth conditions.

The *Anabaena* sp. ATCC 33047 strain was procured from the UTEX culture collection of algae at the University of Texas at Austin (www.utex.org). The strain was isolated from the Texas gulf coast more than 5 decades ago. It was then designated *Anabaena* CA (36, 37) For conjugation experiments, cells were grown in BG11 medium with added nitrate, in shake flasks (∼150 rpm), under 150 μmol photons m^−2^s^−1^ of white light in ambient air at 38°C. For physiological studies, cells were grown in ASP2 liquid medium with or without added nitrate at desired light intensities. The *Anabaena* sp. ATCC 7120 strain was also acquired from the UTEX culture collection and cells were grown in BG11 medium at 50 μmol photons m^−2^s^−1^ of white light in ambient air at 28°C with or without added nitrate.

### Genetic modification and construction of strains.

The *ΔnblA* strain was generated by replacing the *nblA* gene with a kanamycin resistance cassette using homologous recombination. The deletion construct was conjugated into *Anabaena* 33047 using a modified helper plasmid (pSL3348), containing five methylase or methyltransferase genes from the genome of *Anabaena* 33047 (table S1). Details of vector construction and conjugation protocol are provided as supplementary information.

10.1128/mbio.03408-21.7 TABLE S1 Details of the methylase or methyltransferase genes and their upstream regions that were cloned into the newly constructed helper plasmid pSL3348 that was used to successfully conjugate *Anabaena* 33047 and generate the Δ*nblA* mutant. Download Table S1, DOCX file, 0.01 MB. Copyright © 2021 Bandyopadhyay et al. 2021 Bandyopadhyay et al. https://creativecommons.org/licenses/by/4.0/ This content is distributed under the terms of the Creative Commons Attribution 4.0 International license.

### Growth analysis.

For growth measurements, *Anabaena* 33047 cultures were maintained in ASP2 medium with shaking at ∼150 rpm under 250 μmol photons m^−2^s^−1^ of white light in ambient air at 38°C. *Anabaena* 7120 cultures were maintained in BG11 medium with shaking at ∼150rpm under 50 μmol photons m^−2^s^−1^ of white light in ambient air at 28°C. Culture aliquots were then diluted to an OD730 of 0.05 in a Multi-Cultivator MC 1000-OD device (Photon Systems Instruments, Drasov, Czech Republic), and growth under continuous light-emitting diode (LED) light of different intensities in ambient air supplemented with 1% CO_2_ were measured at 730 nm.

### Fluorescence microscopy.

Cells from 24 to 48h liquid culture were imaged using a Nikon Eclipse 80i microscope equipped with a Photometrics Cool Snap ES CCD camera (Roper Scientific). Illumination was provided by a metal halide light source (X-Cite). PBS fluorescence was detected using a 560/40 nm excitation filter, a 595 nm dichroic beam splitter, and a 630/60 nm emission filter.

### Measuring PBS in heterocysts.

Steady-state fluorescence kinetics of WT and Δ*nblA* heterocysts of *Anabaena* 33047 were measured by the fluorescence kinetic microscope (Photon Systems Instruments, Drasov, Czech Republic, www.psi.cz). PBS was excited with green LED light (LZ1-00G100, 530 nm peak). A steady-state PBS fluorescence was separated from exciting light by a dichroic mirror with 562 nm edge wavelength (Semrock 562 nm edge BrightLine) and collected using an emission filter with transmission band 663.5 to 666.5 nm (Semrock 660/13 nm BrightLine). Signals were measured from individual heterocysts and vegetative cells using the 40× objective (Zeiss Plan-Apochromat 40×/1.4 oil). The average signal from a minimum of 10 cells was used to obtain the representative PBS signal from the mutant and the WT. The FluorCam7 software developed by Photon Systems Instruments was used to operate the FKM and analyze the data.

### Nitrogen fixation assay.

Nitrogenase activity was measured using the acetylene reduction assay, as described in reference (38), and expressed in terms of the ethylene produced per mg of chlorophyll. Cultures were grown in ASP2 medium lacking fixed nitrogen in multicultivator tubes under 250 and 2000 μmol photons m^−2^s^−1^ at 42°C in air supplemented with 3% CO_2_. Cells were transferred to airtight 100 mL glass vials and incubated in a 5% acetylene atmosphere under light at 450 μmol photons m^−2^s^−1^ 38°C for 3 h. Gas samples were withdrawn from the vials, and ethylene production was measured using an Agilent 6890N gas chromatograph equipped with a Poropak N column and a flame ionization detector with argon as the carrier gas (38).

### PSI measurements.

P700 concentration in whole cells was determined using a JTS-10 pump-probe spectrometer (BioLogic, France). Cultures grown to mid-exponential-phase were normalized to 5 μg/mL chlorophyll and incubated at 38°C under the light before measurements. For analysis of P700 kinetics, samples were treated with the required inhibitors (10 μM DCMU, 1 μM DBMIB) and dark-adapted for 3 min before subjecting them to an actinic light pulse. The redox state of P700 was monitored by absorption at 705 nm during the 5 min saturating pulse and for 10 s of recovery afterward.

10.1128/mbio.03408-21.8 TEXT S1 Genetic modification and construction of the mutant. Download Text S1, DOCX file, 0.01 MB. Copyright © 2021 Bandyopadhyay et al. 2021 Bandyopadhyay et al. https://creativecommons.org/licenses/by/4.0/ This content is distributed under the terms of the Creative Commons Attribution 4.0 International license.
