# Supplementary figures and images for: Antenna Modification Leads to Enhanced Nitrogenase Activity in a High Light-Tolerant Cyanobacterium
Source: mBio. 2021 Dec 21;12(6):e03408-21. doi: 10.1128/mbio.03408-21 (PMC8689445; doi:10.1128/mbio.03408-21)

Figure S2

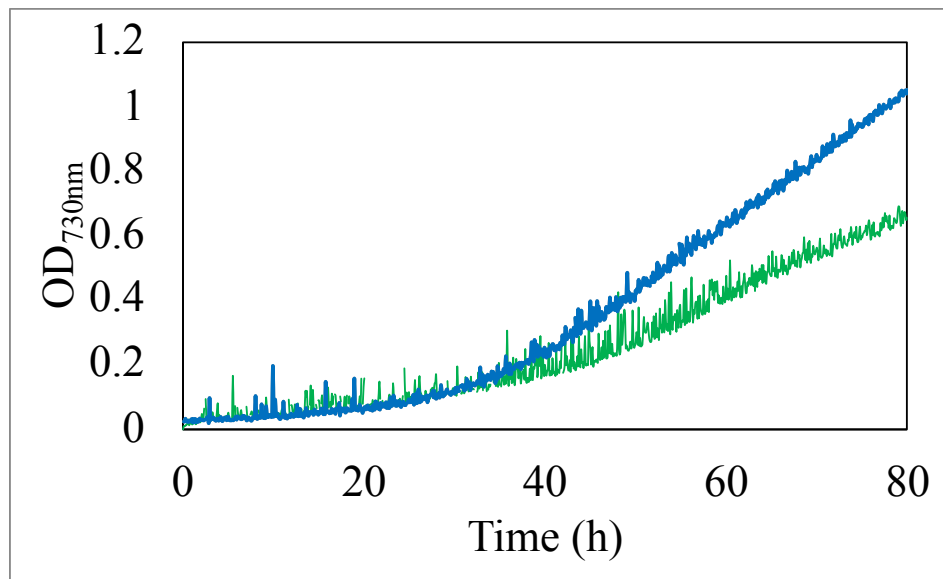

Supplement: FIG S2 [file mbio.03408-21-sf002.pdf]

Figure S1

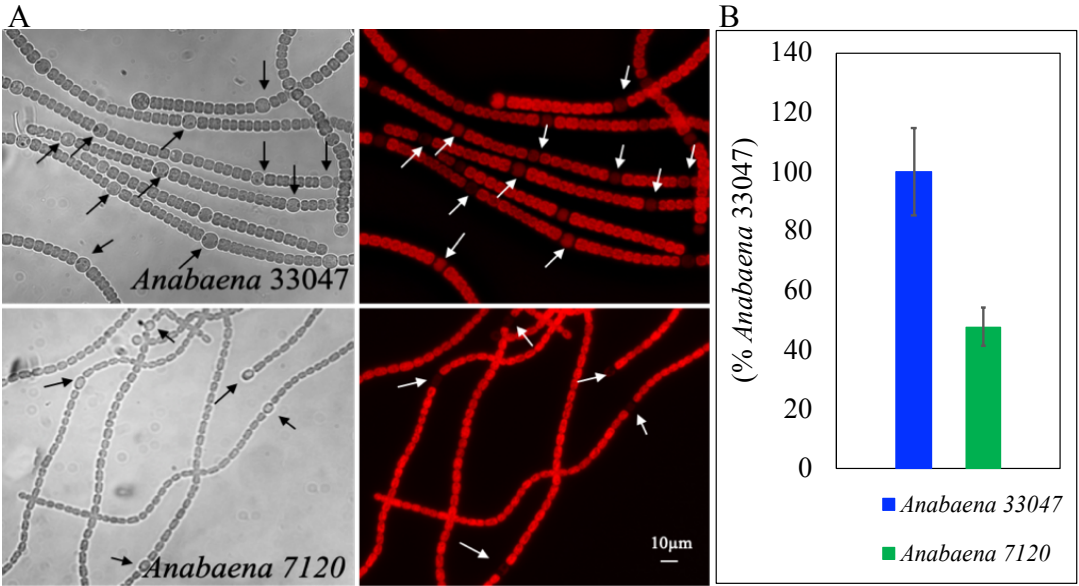

Supplement: FIG S1 [file mbio.03408-21-sf001.pdf]

Figure S3

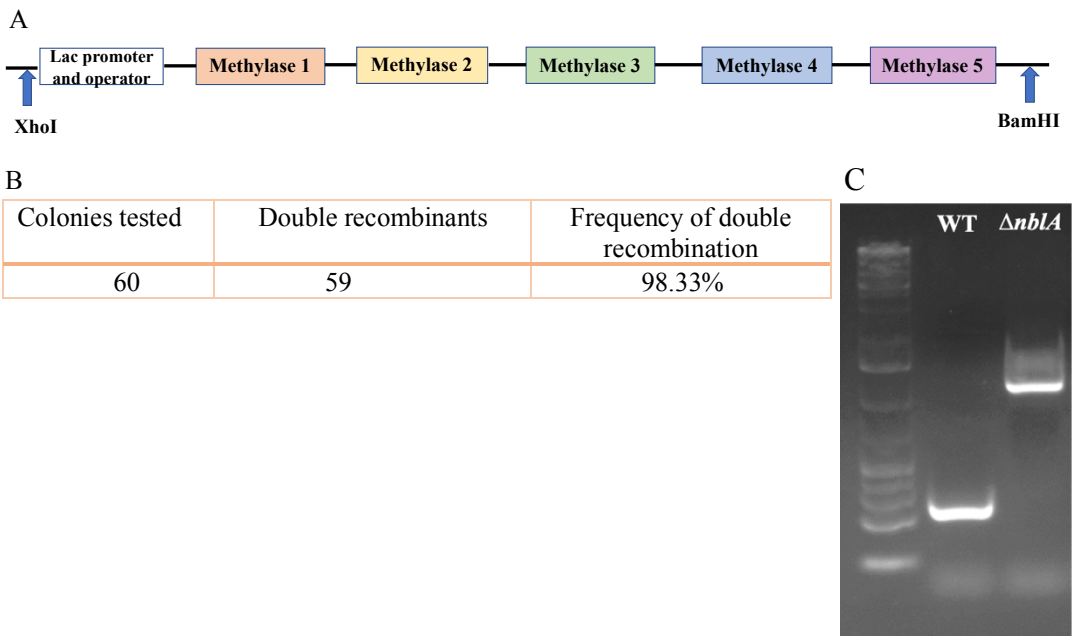

Supplement: FIG S3 [file mbio.03408-21-sf003.pdf]

Figure S4

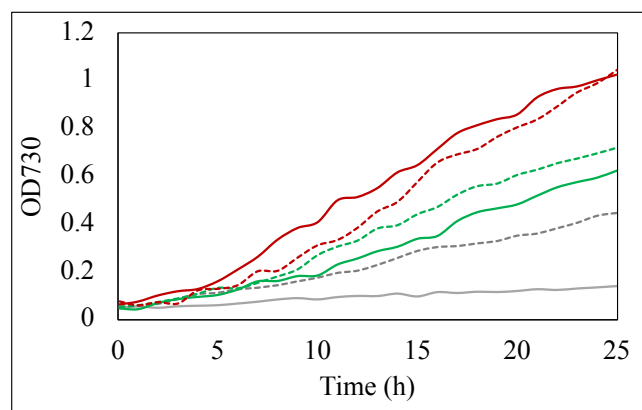

Supplement: FIG S4 [file mbio.03408-21-sf004.pdf]

Figure S5

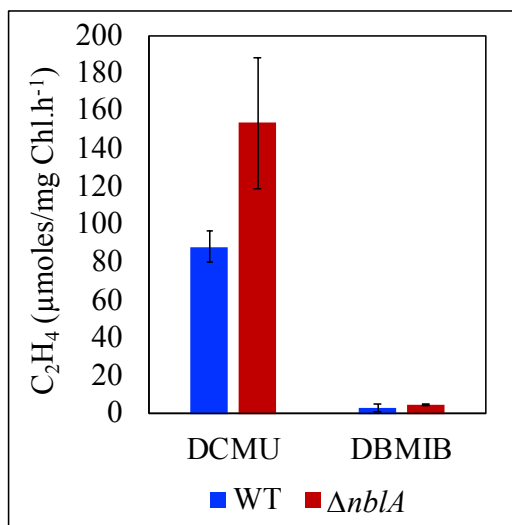

Supplement: FIG S5 [file mbio.03408-21-sf005.pdf]
