# Supplementary material for: Antenna Modification Leads to Enhanced Nitrogenase Activity in a High Light-Tolerant Cyanobacterium
Source: mBio. 2021 Dec 21;12(6):e03408-21. doi: 10.1128/mbio.03408-21 (PMC8689445; doi:10.1128/mbio.03408-21)
Supplement: FIG S6 [file mbio.03408-21-sf006.pdf]

Figure S6

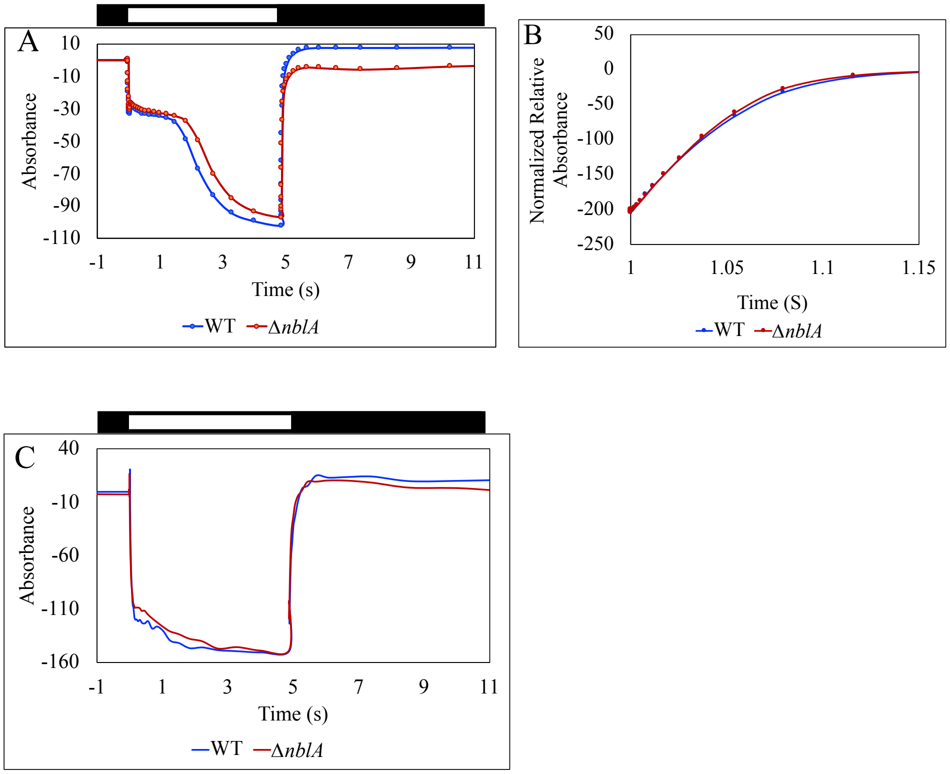

Figure S6

P700 redox kinetics for WT and Δ*nblA* mutant of *Anabaena* 33047 grown under high light (2000 μmol photons $m^{-2}s^{-1}$). (A) P700 kinetics in the WT and Δ*nblA* mutant in the absence of any inhibitor. (B) The details of the re-reduction kinetics of $P700^{+}$ in the dark (normalized to total oxidizable P_{700}) for the experiments in figure 4B. (C) P700 kinetics in the WT and Δ*nblA* mutant grown under nitrogen sufficient conditions and assayed in the presence of DCMU (10μM). Each trace is an average of 3 independent experiments.
