# Supplementary material for: Antenna Modification Leads to Enhanced Nitrogenase Activity in a High Light-Tolerant Cyanobacterium
Source: mBio. 2021 Dec 21;12(6):e03408-21. doi: 10.1128/mbio.03408-21 (PMC8689445; doi:10.1128/mbio.03408-21)
Supplement: TABLE S1 [file mbio.03408-21-st001.docx]

Table S1

| IMG JGI ID | NCBI ACCESSION | Upstream region included |
| --- | --- | --- |
| 2725875708 | WP_066376493.1 | 32bp |
| 2725876806 | WP_066378619.1 | 236bp |
| 2725877114 | WP_066379376.1 | 144bp |
| 2725878383 | WP_084386927.1 | 242bp |
| 2725878968 | WP_066383088.1 | 92bp |

Details of the Methylase or methyl transferase genes and their upstream regions that were cloned into the newly constructed helper plasmid pSL3348 that was used to successfully conjugate *Anabaena* 33047 and generate the ∆nblA mutant.
