# Supplementary material for: Antenna Modification Leads to Enhanced Nitrogenase Activity in a High Light-Tolerant Cyanobacterium
Source: mBio. 2021 Dec 21;12(6):e03408-21. doi: 10.1128/mbio.03408-21 (PMC8689445; doi:10.1128/mbio.03408-21)
Supplement: TEXT S1 [file mbio.03408-21-s0001.docx]

Supplementary information

**Materials and Methods**

Genetic modification and construction of mutant

The suicide plasmid pRL271-*nblA* was constructed by cloning ~1.5kb fragments upstream and downstream of the *nblA* gene into the *XhoI* and *BglII* sites in pRL271. A kanamycin resistance cassette was inserted between them. Cloning was performed in *E. coli* XL1-blue strain on LB-agar with 20μg/mL chloromycetin or 50μg/mL kanamycin. Triparental conjugation was performed using pRL443 as the conjugal plasmid and a newly constructed plasmid pSL3348 as the helper plasmid, with pRL271-*nblA* transformed into the HB101 strain already containing pSL3348. Overnight cultures of the *E. coli* strains were mixed with *Anabaena* 33047 cultures grown in BG11 medium adjusted to an OD730 of 0.8 and sonicated for 10min to get rid of clumping. Mixed cells were incubated together for 6h under low light at 30°C and then were plated onto HATF08250 filters (Millipore-Sigma, St Louis, MO) on BG11 plates supplemented with 5% (vol/vol) Luria Broth. After 48h incubation at 38°C under 150μmol photons m^-2^s^-1^ light, the conjugation filters were transferred onto BG11 plates supplemented with 20μg/ml neomycin. Neomycin resistant colonies appeared within one week of conjugation.

To construct the helper plasmid pSL3348, five methylase or methyltransferase genes were selected from the genome of *Anabaena* 33047. They were designed to be driven by the native or lac promoters and then cloned into the *XhoI* and *BglII* sites in pRL623. The five genes were cloned with along with their upstream regions (Supplementary table 1) to facilitate efficient ribosome binding. All the DNA fragments were ligated using the Gibson Assembly system (Promega).
